# Synchronous Improvement of Mechanical and Damping Properties of Structural Damping Composites with Polyetherimide Non-Woven Fabric Interlayers Loaded with Polydopamine and Carbon Nanotubes

**DOI:** 10.3390/polym15143117

**Published:** 2023-07-21

**Authors:** Shihao Zhou, Yuanchang Lai, Junchi Ma, Bin Liu, Nannan Ni, Feng Dai, Yahong Xu, Zhaodi Wang, Xin Yang

**Affiliations:** 1 Key Laboratory for Light-Weight Materials, Nanjing Tech University, Nanjing 210009, China201910006627@njtech.edu.cn (F.D.); 201910006628@njtech.edu.cn (Z.W.); 2 Beijing Institute of Aerospace Systems Engineering, Beijing 100076, China

**Keywords:** structural damping composites, loss factor, PEI non-woven fabrics, carboxylated carbon nanotubes, polydopamine

## Abstract

Structural damping composites exhibit considerable potential in aerospace and other fields due to their excellent damping and vibration reduction performance, as well as their structural carrying capacity. However, conventional structural damping composite materials generally do not combine excellent mechanical and damping properties at the same time, which makes it difficult for them to meet the practical demand in engineering. In this paper, polyetherimide (PEI) non-woven fabric interlayer materials loaded with quantified polydopamine (PDA) and carboxylated multi-walled carbon nanotubes (MWCNTs-COOH) were used to prepare carbon fiber-reinforced bismaleimide composites through the co-curing process. The mechanical and damping properties of the composites were systematically studied. The results demonstrate that PEI non-woven fabric interlayers loaded with PDA and MWCNTs-COOH can synchronously improve the mechanical and damping properties of the co-cured composites. The incorporation of carbon nanotubes and polydopamine during the co-curing process synergistically improves the flexural strength, flexural modulus, interlaminar shear strength, and impact fracture toughness of the composites. Most importantly, damping properties show an increase of 45.0% in the loss factor of the co-cured composites. Moreover, the reinforcement mechanism was investigated using the optical microscopy and scanning electron microscopy, which indicated that the PEI interlayers loaded with carbon nanotubes and polydopamine form a rich resin area between the layers of the composites.

## 1. Introduction

In recent years, the demand for vibration control in aerospace vehicles has increased with their increasing maneuverability and flight speed. Traditional viscoelastic damping materials have limitations, such as low mechanical strength, aging and failure, and delamination, that cannot meet the requirements [1]. Co-cured composites that combine structural bearing and damping vibration reduction capabilities have become a main trend in the development of composites [2]. The commonly used co-curing additives are viscoelastic materials, such as viscoelastic rubber. However, due to the relatively low glass transition temperature, these types of additives usually lead to a sharp decrease in the elastic modulus and heat resistance of the composites [1]. Therefore, the use of high damping viscoelastic materials between the composite layers will lead to a general decrease in the mechanical properties of the prepared composites by more than 40% [3]. Therefore, new interlayer materials and damping energy dissipation mechanisms need to be developed for the preparation of co-cured composites.

Researchers have made efforts to improve the damping performance of co-cured composites using different methods [4,5,6,7]. Ni et al [8]. inserted aramid non-woven fabric loaded with PVDF between composite layers, which significantly improved the damping performance and fracture toughness of the composites. The basic damping mechanism is that the thermoplastic interlayer materials form a resin-rich region with a high loss factor between the layers of the composites during co-curing, increasing the loss factor by 108.0%. McLaughlin et al [9]. added polyetheretherketone (PEI) film layers to carbon fiber-reinforced polyetheretherketone (CF/PEEK) laminates and showed that the addition of PEI films improved the type I interlaminar fracture toughness (G_IC_) and the type II interlaminar fracture toughness (G_IIC_). However, the above literature mainly improves the damping performance by introducing new damping materials into the composite materials. One significant weakness of this kind of sandwich-like structure is its huge thickness due to the addition of interlayers, which decreased mechanical properties of the composite [2].

In addition, nanomaterials were also reported to be able to improve the composite damping and mechanical properties simultaneously [10,11,12]; however, their poor dispersion and high cost greatly limit their large-scale application. Joy et al [13]. reported a high-damping composite based on epoxy reinforced with multi-walled carbon nanotubes (MWCNTs) and modified with an adduct of epoxy and CTBN. Significant improvement in vibration damping is observed beyond a certain level of adduct content. A unique microstructure formation due to the reaction induced phase separation in the epoxy/adduct/CNT composite results in peak damping behavior. Kim et al [14]. located the highly arranged carbon nanotube yarns treated with non-ionic surfactants at the interlayers and oriented along the loading direction, which provides excellent damping and stiffness characteristics. Ouyang et al [15]. designed a new type of carbon nanotube (CNT)-modified traditional thermoplastic porous film, which was interspersed at the interface of carbon fiber-reinforced plastic (CFRP). The composites exhibited good damping properties and reasonable mechanical properties. However, the nanomaterials tend to aggregate, which further damages the internal structure of the composite materials.

In order to combine mechanical and damping properties at the same time, a series of new structural damping composites were prepared by incorporating polyetherimide non-woven fabric with good heat resistance, high strength and modulus, high porosity, and low density as interleaved materials into CFRP [16]. The high porosity of the polyetherimide non-woven fabric does not cause resin flow blockage during curing, thus avoiding excessive thickness increase in the composite materials. To further improve the mechanical and damping performance of the composites, carboxylated carbon nanotubes were loaded onto the polyetherimide non-woven fabric [17,18], and polydopamine-modified carbon nanotubes were used to improve their surface chemical activity and interface bonding strength, thereby improving the agglomeration issue of the nanomaterials.

## 2. Experimental

### 2.1. Materials

The polyetherimide non-woven fabrics had an areal density of 5.0 g/m^2^ and were purchased from Bludotasia Engineering Yangjiang Ltd. (Yangjiang, China) MWCNTs-COOH purchased from Suzhou Zhaojia New Material Co., Ltd. (Suzhou, China). Dopamine hydrochloride was purchased from Aladdin Reagent Co. (Ontario, CA, USA). Tris-Hydrochloride Buffer (1 M, pH = 8.5) was purchased from Aladdin Reagent Co. ZT7G/SQ6419 prepreg was purchased from Jiangsu Sanqiang Composites Co. (Xuzhou, China).

### 2.2. Preparation of MWCNTs/PEI Structural Damping Interlayer Material

**Figure 1:**
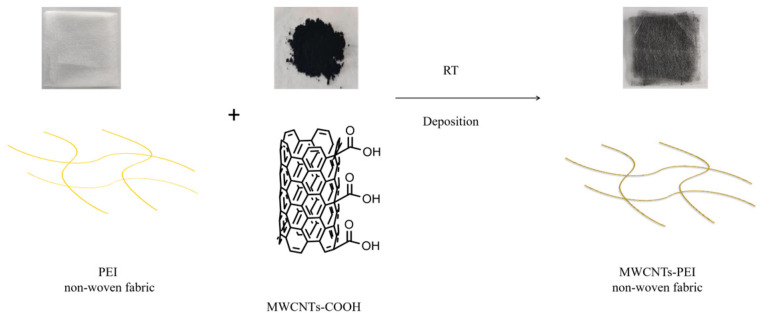
Schematic illustration of preparation process of MWCNTs-PEI.

The MWCNTs/PEI structural damping interlayer materials were prepared using a solution impregnation method. Initially, 2 wt% of MWCNTs-COOH were uniformly dispersed in anhydrous ethanol by ultrasonication for 12 h. Next, the PEI non-woven fabrics were impregnated with the MWCNTs-COOH dispersion for 48 h to obtain the MWCNTs/PEI structural damping interlayer materials. Subsequently, the MWCNTs/PEI structural damping interlayer materials were rinsed with deionized water to remove excess ethanol. Finally, the MWCNTs/PEI structural damping interlayer materials were dried under a high vacuum at 90 °C for 12 h to evaporate the excess water. The process is shown in Figure 1.

### 2.3. Preparation of MWCNTs@PDA/PEI Structural Damping Interlayer Material

**Figure 2:**
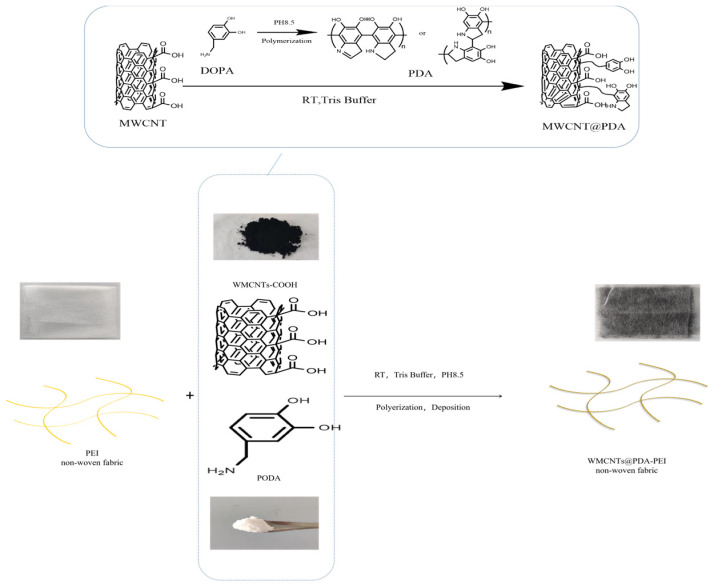
Schematic illustration of preparation process of MWCNTs@PDA-PEI.

Dopamine was added to 100 mL Tris buffer solution (100 mM, pH = 8.5) to prepare 1 wt% dopamine hydrochloride monomer solution, and then MWCNTs/PEI structural damping interlayer materials were immersed in the solution. The dopamine monomer will polymerize on the MWCNTs/PEI structural damping interlayer materials for 48 h to obtain the MWCNTs @ PDA/PEI structural damping interlayer materials. Then the MWCNTs@PDA/PEI structural damping interlayer materials were washed with deionized water to remove any unreacted dopamine hydrochloride monomer. Finally, the MWCNTs@PDA/PEI structural damping interlayer materials were dried under a high vacuum at 90 °C for 12 h to evaporate the excess water. The process is shown in Figure 2 [19,20].

### 2.4. Preparation of Three Structural Damping Composites

**Figure 3:**
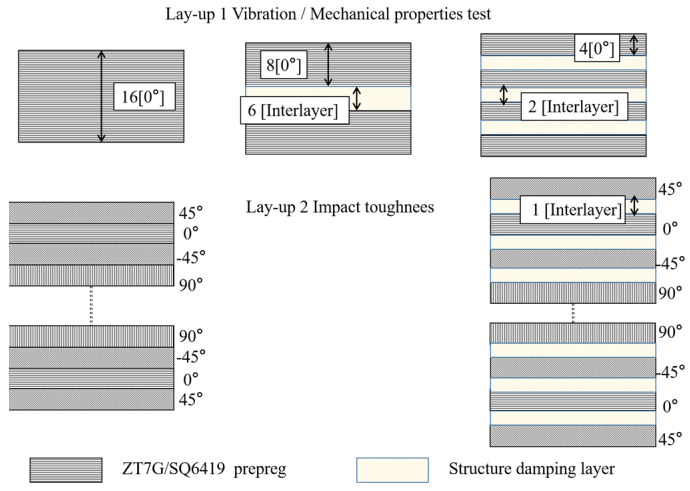
Schematic illustration of the co-cured composites.

Three structural damping composites were prepared by inserting three different damping interlayer materials, including the PEI non-woven fabrics, MWCNTs/PEI structural damping interlayer materials, and MWCNTs@PDA/PEI structural damping interlayer materials, between the ZT7G/SQ6419 prepreg, respectively. The lay-up of the composites was determined based on the design shown in Table 1 and Figure 3. The curing process was carried out in an auto lave, following the procedures listed in Table 2.

### 2.5. Characterization

To measure the chemical composition of the modified MWCNTs, X-ray photoelectron spectroscopy measurements (XPS, Thermo Fisher Nexsa, Thermo Fisher Scientific, Waltham, MA USA) were carried out on modified MWCNTs. Fourier transform infrared (FTIR, Nicolet IS50, Thermo Fisher Scientific) spectroscopy was used to investigate the organic functional groups of modified MWCNTs in the mid-infrared region (4000−500 cm^−1^). Differential scanning calorimetry (DSC) analysis was performed on a DSC250 TA instrument in a nitrogen atmosphere with a heating rate of 10 °C/min and a temperature range of 30–300 °C, and all samples were dried at 80 °C for 12 h before testing.

The static mechanical properties test was carried out for the co-cured composite with Instron 3382 material testing machine. The size of each sample for the flexural performance test was 100 mm × 12.5 mm according to GB/T3356 (Chinese standard), and the loading rate of the flexural performance test was 2 mm/min, and 6 samples were tested for each composite system. The size of each sample for the interlaminar shear strength test was 20 mm × 6 mm according to GB/T3357 (Chinese standard), and 6 samples were tested for each composite system. For the impact test, the sample was subjected to an impact power load using a ZBC 8400-B impact testing machine. The size of the sample was 60 mm × 120 mm according to GB/T 1451 (Chinese standard), and 6 samples were tested for each composite system.

The Dynamic Mechanical Analyzer (DMA Q800, TA Instruments, New Castle, DE, USA) was performed under N_2_ atmosphere with a heating rate of 5 °C/min from 30 °C to 300 °C in 3-point bending mode with a strain amplitude maintained at 0.008%, and a frequency of 1 Hz [21]. The sample dimension was 60 mm × 10 mm in size, and 6 samples were tested for each composite system.

Single cantilever free vibration decaying tests were carried out for the co-cured composite with the LMS SCADAS Mobile SCM01 (Wuhan Aerospace Star Technology Co., LTD, Wuhan, China). Single cantilever free vibration decaying tests were performed according to ASTM E756-05. The sample dimension used was 180 mm × 10 mm. In addition, the fixed length was 30 mm in the single cantilever free vibration decaying test, and 6 samples were tested for each composite system.

The morphologies of the composite samples were observed with a scanning electron microscope (TESCAN CLARA, Brno, Czech Republic) at an operating voltage of 15 KV. All samples were gold plated prior to testing.

## 3. Results and Discussions

### 3.1. Characterization of MWCNTs and MWCNTs@PDA

Figure 2 illustrates the process of preparing MWCNTs@PDA through dopamine self-polymerization. This process occurs spontaneously under oxidative and alkaline conditions, resulting in a product with high adhesion properties that can easily adhere to solid surfaces. The benzene ring in PDA can also interact with MWCNTs through π–π bonding interactions, enabling the coating of the MWCNTs surface with PDA [22].

**Figure 4:**
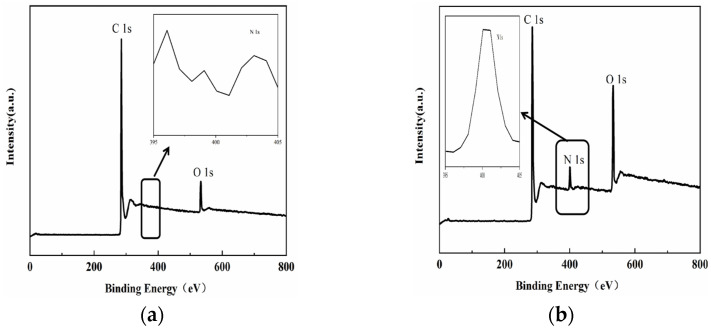
XPS spectra of (**a**) MWCNTs and (**b**) MWCNTs @PDA.

**Figure 5:**
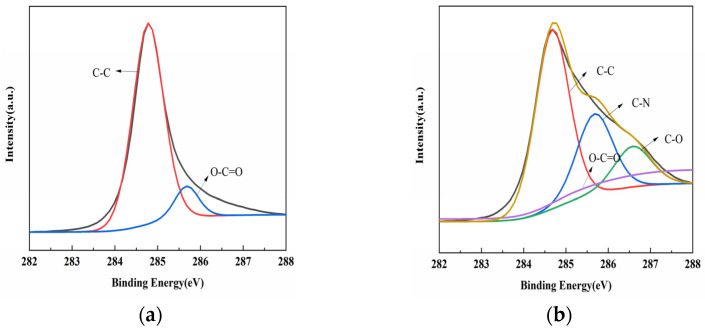
XPS C1s peak-fitting curves of (**a**) MWCNTs and (**b**) MWCNTs @PDA.

The chemical composition of the modified MWCNTs was analyzed using X-ray photoelectron spectroscopy (XPS). Figure 4a shows the wide-scan XPS spectra of the MWCNTs–COOH, which exhibit two peaks corresponding to C1s and O1s. The C1s XPS spectrum in Figure 5a is separated into two carbon bonds, namely C–C (284.6 eV) and O–C=O (285.5 eV). In contrast, the MWCNTs@PDA spectrum shows an increased N1s peak due to the presence of the modifying agent PDA (Figure 4b). The C1s XPS spectrum of MWCNTs@PDA (Figure 5b) shows the appearance of C–N (285.5 eV) and C–O (286.5 eV) bonds, indicating successful modification with PDA [23].

**Figure 6:**
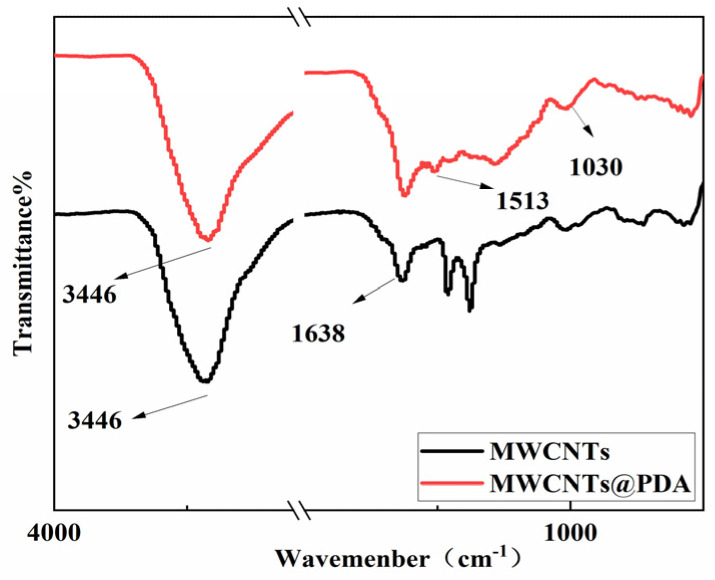
FT-IR spectra of MWCNTs and MWCNTs @PDA.

In order to further detect the groups in modified MWCNTs, FTIR spectra were analyzed, and the results are shown in Figure 6. The characteristic bands observed at 3446 cm^−1^ and 1638 cm^−1^ are attributed to the –OH and C–O vibration, respectively. However, comparing the spectrum of carboxylated MWCNTs and MWCNTs@PDA, it can be found that the peak at 3446 cm^−1^ is significantly stronger and deeper, which is caused by –OH and –NH from the polydopamine coated on the carbon nanotubes after modification [24]. The peak at 1030 cm^−1^ corresponds to the characteristic absorption peak of the C–N bond of PDA, and the peak at 1513 cm^−1^ corresponds to the N-H shear vibration peak on the surface of PDA [19].

### 3.2. Mechanical Properties of the Structural Damping Composites

#### 3.2.1. Static Mechanical Properties of the Structural Damping Composites

The flexural strength, flexural modulus, and interlaminar shear strength of the control sample and co-cured composite are shown in the supplementary data (Tables S1–S7, and Figures S1–S14); the values are listed in Table 3. The flexural strength of the control composite is 1724.95 MPa, the flexural modulus is 123.38 GPa, and the interlaminar shear strength is 103 MPa. The fiber volume fraction is approximately 60%, and the thickness is 1.99 mm. The thickness of the co-cured composite has slightly increased by about 80–100 μm due to the addition of interlayers. The PEI non-woven fabric composite with a concentrated interlayer structure reduces the flexural strength, flexural modulus, and interlaminar shear strength by 6.68%, 8.54%, and 3.29%, respectively, compared to the control composite. The flexural strength, flexural modulus, and interlaminar shear strength decreased by 10.95%, 9.09%, and 7.99% for PEI non-woven fabric composite with a dispersion interlayer structure. The flexural strength of MWCNTs/PEI composite with a concentrated interlayer structure is 1677.90 MPa, the flexural modulus is 126.97 GPa, and the interlayer shear strength is 101.46 MPa. The flexural modulus of MWCNTs/PEI composite with a concentrated interlayer structure is slightly increased compared with the control sample. Furthermore, the mechanical properties of both the concentrated interlayer composite and dispersed interlayer composite are improved after PDA modification. The flexural strength of the MWCNTs@PDA/PEI composite with concentrated interlayers reaches 1888.80 MPa, the flexural modulus is 141.84 GPa, and the interlaminar shear strength reaches 110.29 MPa, which is increased by 9.50%, 14.96%, and 7.08%, respectively. The MWCNTs@PDA/PEI composite with the dispersed interlayers is not as good as the MWCNTs@PDA/PEI composite with the concentrated interlayers. The interlaminar flexural strength of the MWCNTs@PDA/PEI composite with the dispersed interlayers is 1867.23 MPa, and the flexural modulus of 107.17 MPa, and the interlaminar shear strength of 107.17 MPa, respectively, an increase of 8.25%, 7.85%, and 4.05%, respectively.

In summary, the flexural strength, flexural modulus, and interlaminar shear strength of the MWCNTs@PDA/PEI composite do not decrease significantly. The MWCNTs@PDA/PEI composite exhibits the best mechanical properties, followed by the MWCNTs/PEI, while the PEI interlayer composite has the poorest effect. From the perspective of the interlayer insertion method, the concentrated interlayer insertion method exhibits better mechanical properties than the separated interlayer insertion method.

#### 3.2.2. Dynamic Mechanical Properties of Structural Damping Composites

**Figure 7:**
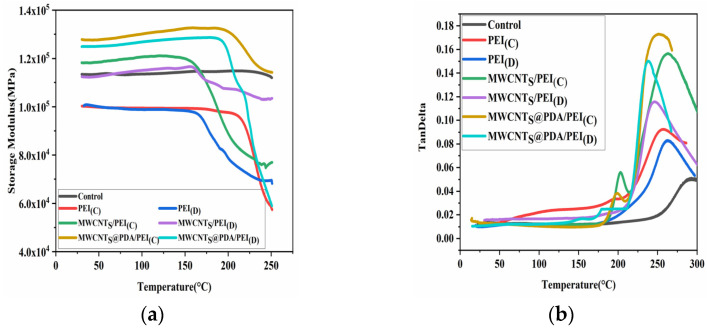
Storage modulus (**a**) and loss factor (**b**) of co-cured composites.

**Figure 8:**
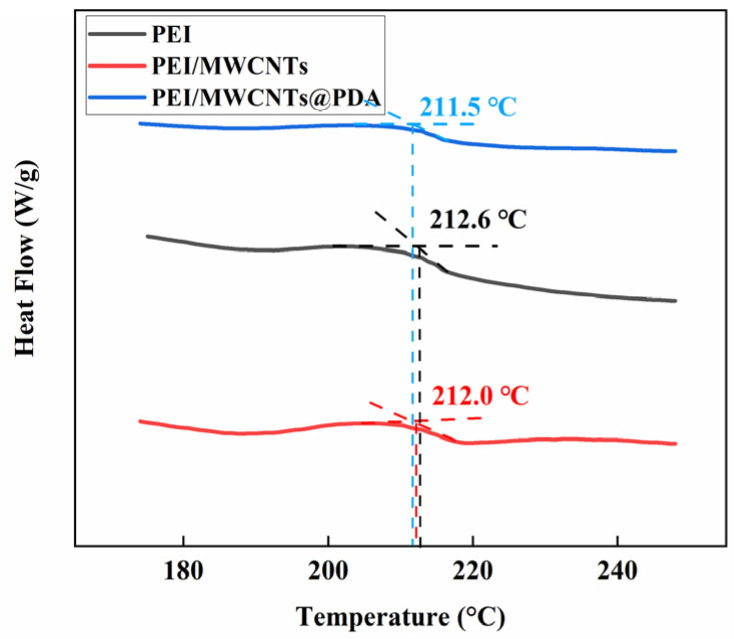
Differential scanning calorimetry curves of PEI non-woven fabric, PEI/MWCNTs non-woven fabric, and PEI/MWCNTs@PDA non-woven fabric.

Figure 7a,b show the storage modulus and loss factor of different co-cured composites with temperature, respectively. It can be seen in Figure 7a that the storage modulus of the control sample is about 113 GPa at room temperature and remains constant until 250 °C. However, all of the co-cured composites show a significant decrease until 250 °C, indicating that the addition of the interlayer material lowers the glass transition temperature of the composite. The initial storage modulus of the PEI non-woven fabric composite inserted is slightly lower than that of the control sample, which is about 100 GPa. The storage modulus of the MWCNTs@PDA/PEI composite is higher than the control sample. The highest storage modulus is the MWCNTs@PDA/PEI composite with concentrated interlayers, which reached about 127 GPa, about 12.4% higher than that of the control sample. In addition, the storage modulus and the glass temperatures of the concentrated interlayer composite are slightly higher than those of the dispersed interlayer composite. This indicates that the mechanical properties of the sample vary greatly depending on the location of the intercalated layers at high temperatures [25]. As shown in Figure 7b, the loss factor of the control composite is about 0.015 before 200 °C, the peak loss factor is about 0.051, and the glass transition temperature of 292 °C. The glass transition temperatures of the composite with different co-curing are decreased, and the loss factors and glass transition loss peaks are higher than those of the control sample. The loss factor of MWCNTs@PDA/PEI reached the highest level of 0.18, which is 253% higher than the control sample. It is noteworthy that the loss factor peak appears in all co-cured composites around 200 °C, and the loss factor peak is more obvious in the sample with concentrated interlayers. The reason for this is that, as shown in Figure 8’s DSC curves of three types of non-woven fabrics, the glass transition occurs in PEI non-woven fabric around 212 °C.

#### 3.2.3. Impact Toughness Properties of the Structural Damping Composites

**Figure 9:**
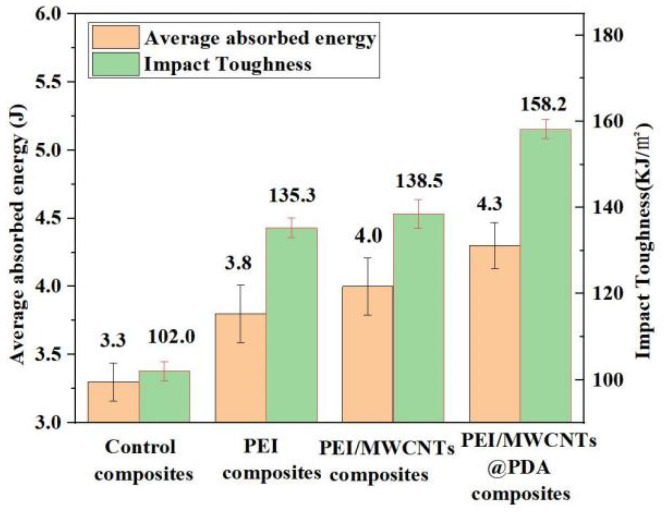
Average absorbed energy and impact toughness of co-cured composites.

**Figure 10:**
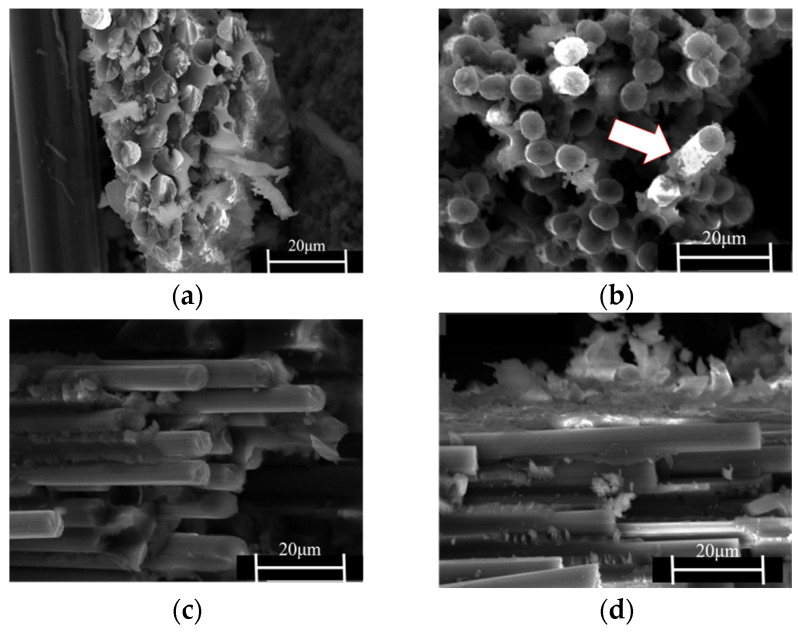
SEM images of the fracture interface of (**a**) control sample, (**b**) co-cured composite with PEI, (**c**) co-cured composite with PEI/MWCNTs, (**d**) co-cured composite with PEI/MWCNTs@PDA.

The average absorbed energy and impact toughness of the co-cured composite are presented in Figure 9. The impact toughness of the control composite is measured to be 102 KJ/m^2^ with an absorbed energy of 3.3 J. Figure 10 shows the scanning electron micrographs of the fracture surface of the composite material. In Figure 10a, the carbon fibers display relatively neat fracture, indicating brittle fracture. This means that only a small amount of energy can be absorbed by the fracture of fibers and resin, indicating that the sample cannot dissipate vibration energy during vibration [26].

The absorbed energy of the PEI non-woven fabric composite increased to 3.8 J and the impact toughness was measured to be 135.3 KJ/m^2^. The absorbed energy of the MWCNTs/PEI composite reached 4 J, and the impact toughness is 138.5 KJ/m^2^, which is 35.29% higher than that of the control sample. Furthermore, the impact toughness of the MWCNTs@PDA/PEI composite increased to 158.2 KJ/m^2^, which is 55.09% higher compared to the control sample.

**Figure 11:**
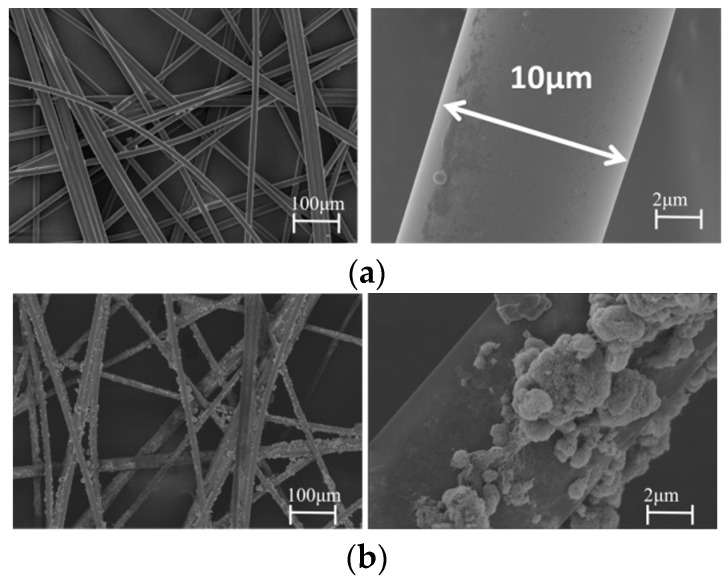

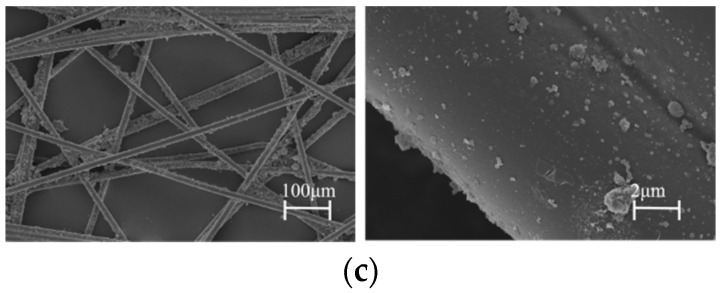
SEM images of (**a**) PEI non-woven fabric, (**b**) PEI/MWCNTs non-woven fabric and (**c**) PEI/MWCNTs@PDA non-woven fabric.

In Figure 11a, the micro-morphology of the high-porosity non-woven fabric is shown. The high porosity of the non-woven fabric allows for the matrix resin to form a continuous structure, ensuring good mechanical strength for the composite. Figure 10b presents a cross-sectional photograph of the PEI non-woven fabric composite, which shows the presence of resin-rich layers (white arrow) between the carbon fiber layers. This phenomenon results in the formation of a bi-continuous structure between the thermoplastic PEI and the matrix resin, which leads to an increased absorbed energy during impact processes. For the MWCNTs/PEI composite material (Figure 10c,d), due to the high specific modulus and strength, high aspect ratio, and high specific surface area of MWCNTs, the “bridging effect” formed by MWCNTs in the resin can hinder the resin from fracturing under impact. The figure also shows a large amount of fiber pull-out in addition to the resin-rich layer [20,27]. However, as shown in Figure 11b, the MWCNTs are not uniformly dispersed on the PEI non-woven fabrics and show significant agglomeration, which reduces the reinforcing effect. Figure 11c depicts the dispersion of PDA-modified MWCNTs on PEI non-woven fabrics, which are more uniformly dispersed with less agglomeration phenomenon. This is because the PDA-modified carbon nanotubes contain a large number of amino and catechol groups. These groups can form strong hydrogen bonds with all organic and inorganic substances, thus improving the interfacial bonding strength and indirectly improving their impact toughness [28,29]. It has been shown that the MWCNTs also have a synergistic effect with PDA to improve the stress transfer efficiency from the PEI matrix to the carbon fiber [30].

**Figure 12:**
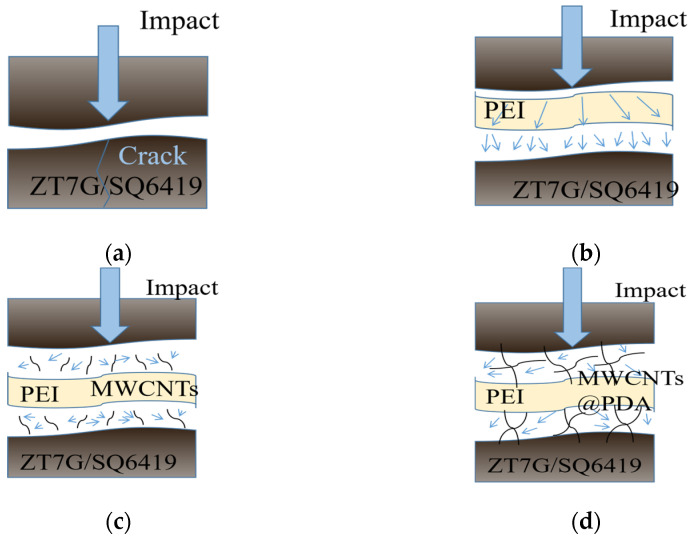
Scheme of the impact test model, (**a**) control sample, (**b**) co-cured composite with PEI, (**c**) co-cured composite with PEI/MWCNTs, (**d**) co-cured composite with PEI/MWCNTs@PDA.

Figure 12 illustrates the impact test model scheme. In the case of the control sample (Figure 12a), cracks induced by impact loading directly affect the weakest layers of the composite. In the PEI composite, such as that shown in Figure 12b, the soft component (PEI non-woven fabrics) can effectively disperse the impact and reduce crack extension in the fibers and interlaminar regions. In the MWCNTs/PEI composite (Figure 12c), MWCNTs can release stress concentrations and prevent crack expansion [31]. Additionally, PDA can enhance the attachment of MWCNTs to the resin, leading to more effective force dispersion, greater energy consumption, and improved impact strength of the composite [32].

### 3.3. Damping Properties of the Structural Damping Composites

**Figure 13:**
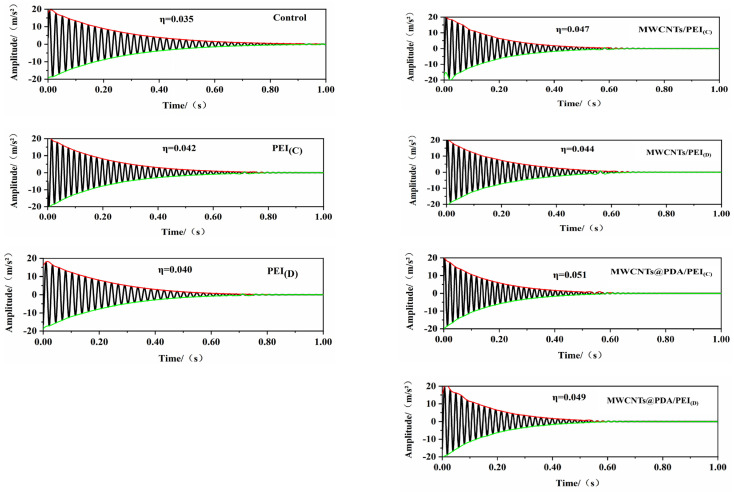
Free vibration decaying curve of co-cured composites.

Figure 13 shows the vibration decaying curves of the co-cured composite obtained from the single cantilever beam vibration test, and the damping factors are derived from Equations (1) and (2).

(1) $$\delta =\frac{1}{n}\ln\frac{A_{n+1}}{A_{1}}$$

(2) $$\eta =\frac{1}{\pi }\delta$$

In the equation, $A_{1}$ and $A_{n+1}$ are the initial and the (n + 1)th amplitude, respectively, where n is the number of cycles, δ is the logarithmic decrement, η is the damping factor, and ξ is the damping ratio. In this article, the logarithmic decrement is calculated for all cycles within 0 s–0.6 s [1,33].

The results from the single cantilever beam vibration test are presented in Figure 13. It can be observed that the damping factor of the control sample is 0.035. The addition of PEI non-woven fabric to the composite increased the damping factor by 20% and 14.28%, respectively. The MWCNTs/PEI composite shows better damping results, owing to the frictional damping effect between the MWCNTs and the matrix material. The damping factor of MWCNTs/PEI composite with a concentrated intermediate layer is 0.047, which increased by 25.53%. In addition, the MWCNTs/PEI composite with a dispersed interlayer has a damping factor of 0.044, which increased by 25.71%. The damping factors of the MWCNTs@PDA/PEI composite are further improved, reaching 0.051 and 0.049, respectively, which corresponded to an increase of 45.71% and 40% compared to the control sample. These results are consistent with the trend observed in the DMA tests.

### 3.4. Structural Analysis of the Structural Damping Composites

Analysis of the structure of co-cured composite material is crucial to understand their mechanical and damping properties. The use of polyetherimide non-woven fabric loaded with PDA and MWCNTs–COOH has been shown to improve both the mechanical strength and damping performance of the composite. To further improve the damping performance and mechanical properties of the structural damping composite material, it is essential to understand the structural foundation and micro-mechanisms of the co-cured composite material. This structural analysis can provide a basis for material modification and structural design.

**Figure 14:**
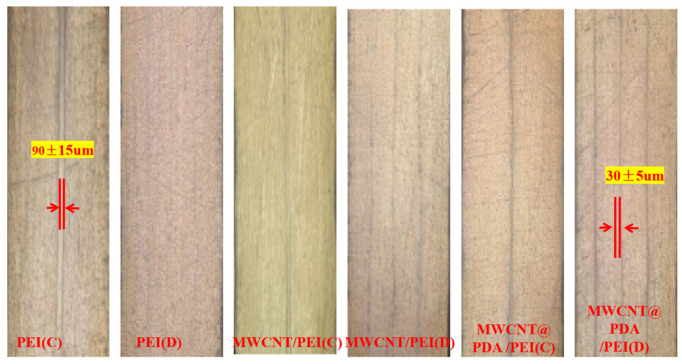
Optical images of co-cured composites.

Figure 14 shows optical micrographs of composite with different interlayers during co-curing. The carbon fibers are evenly distributed throughout the composite material in the absence of interlayers, and the resin interface layer is thin, about 2–3 μm. When the non-woven fabric sandwich is inserted, a resin-rich area with a certain thickness is formed in the insertion area. This area is thicker when the damping material is inserted centrally and thinner when the damping material is inserted decentrally. The differences in the properties of different intercalated composites are mainly related to the differences in the properties of the resin-rich zone between the layers [29,34]. The cured composite material forms a layered composite material with a “hard–soft–hard” microstructure, similar to a traditional constrained damping treatment structure. The carbon fiber layer provides restraint and force transfer, while the resin-rich layer in the middle is the main source of damping energy dissipation [6,35].

**Figure 15:**
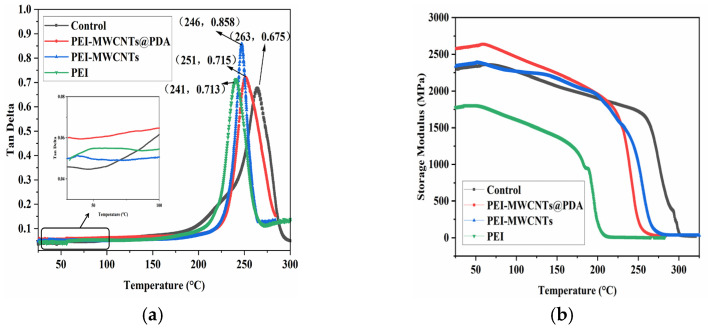
Storage modulus (**a**) and loss factor (**b**) of pure resin SQ6419 and co-curd resin SQ6419 composites.

The matrix resin SQ6419 and the matrix resin SQ6419 with three types of damping materials were prepared and tested for dynamic mechanical properties by simulating the resin-rich zone between the layers, and the results are shown in Figure 15. The glass transition temperature of the matrix resin is 263 °C, with a peak loss factor of 0.675. After incorporating the damping material, the glass transition temperature decreased to around 250 °C, but the loss factor increased. On one hand, adding PEI to the SQ6419 curing resin increased the interfacial friction between PEI and the resin. On the other hand, the rapid increase in the loss factor after the glass transition of PEI indicates that it can be transformed into an amorphous region with high energy dissipation capacity and contributes to a high loss factor [1]. In Figure 15a, the damping factor of the matrix resin SQ6419 is about 0.045 at a room temperature of 25 °C. The loss factor increases to 0.049 and 0.053 with the addition of PEI and MWCNTs/PEI, respectively. The loss factor increases to 0.060 with the addition of MWCNTs@PDA/PEI, which is about 33.3% higher than that of the control sample, as shown. However, the loss factor of the pure resin increases rapidly with increasing temperature, exceeding the loss factor of MWCNTs@PDA/PEI composite at around 100 °C. This indicates that the damping material also plays an obstructive role, hindering the movement of resin chains near Tg, and leading to the degradation of the loss factor [21]. Figure 15b shows the storage modulus of the matrix resin SQ6419 and the matrix resin SQ6419 with three types of damping materials. The lowest storage modulus is inserted into PEI non-woven fabric. The storage moduli of the samples loaded with MWCNTs and MWCNTs@PDA are significantly higher than that of the control sample. This proved that the addition of MWCNTs has a significant strengthening and stiffening effect on the resin; this is consistent with the temperature curve of the storage modulus of the composite.

## 4. Conclusions

In this work, polyetherimide(PEI) non-woven fabric interlayer materials loaded with quantified polydopamine(PDA) and carboxylated multi-walled carbon nanotubes (MWCNTs-COOH) were used to prepare carbon fiber-reinforced bismaleimide composites through the co-curing process. The effect of interlayer materials on the mechanical and damping properties of the resulting composites was investigated and compared. The FTIR and XRD results demonstrated that the MWCNTs and PDA were successfully loaded onto the non-woven fabric interlayer materials. It was found that the inclusion of a PEI non-woven fabric increased the damping factors by 20.00%, while the flexural strength significantly decreased by 12.28%. The associated with the damping properties is attributed to forming resin-rich zones with higher loss factor of the composite, as evidenced by optical images and SEM images. Similarly, the loss factor of MWCNTs/PEI composites further increased due to the MWCNTs/PEI non-woven fabric-formed “bridging effect” within the resin layer. However, MWCNTs/PEI non-woven fabric cannot improve the static mechanical properties and damping properties to the same degree due to the agglomeration of MWCNTs. Compared with the control samples, incorporation of MWCNTs@PDA/PEI non-woven fabric caused a 9.49% increase in flexural strength, from 1724.95 MPa to 1888.80 MPa, and interlaminar shear strength of composite increased from 103.00 MPa to 110.29 MPa. The impact toughness of MWCNTs@PDA/PEI non-woven fabric composite also increased from 102.00 MPa to 158.20 MPa. In addition, the insertion of MWCNTs@PDA/PEI non-woven fabric composite exhibited a high vibration decay rate during the free vibration decay test, and the measured damping factor was 0.051, corresponding to 45.71% improvement compared with that of the control sample. In conclusion, incorporating MWCNTs@PDA/PEI non-woven fabric into the interlayers had been proved to be an effective method to prepare a new co-cured composite combining higher mechanical and damping properties in this paper.

## Figures and Tables

**Table 1:** Layup of co-cured composites with different non-woven fabrics.

Sample	Lay-up 1	Lay-up 2
Control	[0°_16_]	[45°/0°/−45°/90°]_4s_
Centralized	[0°_8_/d_6_/0°_8_]	[45°/d/0°/d/−45°/d/90°]_4s_
Dispersed	[0°_4_/d_2_/0°_4_/d_2_/0°_4_/d_2_/0°_4_]	
0: ZT7G/SQ6419 prepreg d: structural damping interlayer materials		

**Table 2:** Curing system of structural damping co-curing composites.

Temperature/°C	Pressure/N	Time/Min
150	0	30
150	5 × $10^{4}$	60
160	3 × $10^{5}$	10
180	4 × $10^{5}$	30
200	4 × $10^{5}$	240

**Table 3:** List of flexural properties and interlaminar shear strength of co-cured composites.

Sample	Flexural Strength/MPa	Standard Deviation of Flexural Strength	Flexural Modulus/GPa	Standard Deviation of Flexural Modulus	Interlaminar Shear Strength/MPa	Standard Deviation of Interlaminar Shear Strength	Thickness/mm
Control	1724.95	3.19	123.38	1.60	103.00	4.98	1.99
PEI_(C)_	1609.67	5.32	112.84	3.25	99.61	3.50	2.07
PEI_(D)_	1536.21	3.49	111.21	1.02	94.77	3.23	2.07
MWCNTs/PEI_(C)_	1677.90	3.30	126.97	0.87	101.46	4.49	2.07
MWCNTs/PEI_(D)_	1617.23	4.11	119.65	0.30	100.83	2.85	2.08
MWCNTs@PDA/PEI_(C)_	1888.80	6.41	141.84	4.46	110.29	2.43	2.08
MWCNTs@PDA/PEI_(D)_	1867.23	3.16	133.06	2.16	107.17	4.55	2.09

(C): Centralized (D): Dispersed.

## Data Availability

The data presented in this study are available on request from the corresponding author.

## Supplementary Materials

The following supporting information can be downloaded at: https://www.mdpi.com/article/10.3390/polym15143117/s1, Figure S1: Force-displacement curve of flexural test of control; Figure S2: Force-displacement curve of interlaminar shear strength test of control; Figure S3: Force-displacement curve of flexural test of PEI_(C)_; Figure S4: Force-displacement curve of interlaminar shear strength test of PEI_(C)_; Figure S5: Force-displacement curve of flexural test of PEI_(D)_; Figure S6: Force-displacement curve of interlaminar shear strength test of PEI_(D)_; Figure S7: Force-displacement curve of flexural test of MWCNTs/PEI_(C)_; Figure S8: Force-displacement curve of interlaminar shear strength test of MWCNTs/PEI_(C)_; Figure S9: Force-displacement curve of flexural test of MWCNTs/PEI_(D)_; Figure S10: Force-displacement curve of interlaminar shear strength test of MWCNTs/PEI_(D)_; Figure S11: Force-displacement curve of flexural test of MWCNTs@PDA/PEI_(C)_; Figure S12: Force-displacement curve of interlaminar shear strength test of MWCNTs@PDA/PEI_(C)_; Figure S13: Force-displacement curve of flexural test of MWCNTs@PDA/PEI_(D)_; Figure S14 Force-displacement curve of interlaminar shear strength test of MWCNTs@PDA/PEI_(D)_; Table S1: All data of flexural test and Interlaminar Shear test of control; Table S2: All data of flexural test and Interlaminar Shear test of PEI_(C)_; Table S3: All data of flexural test and Interlaminar Shear test of PEI_(D)_; Table S4: All data of flexural test and Interlaminar Shear test of MWCNTs/PEI_(C)_; Table S5: All data of flexural test and Interlaminar Shear test of MWCNTs/PEI_(D)_; Table S6: All data of flexural test and Interlaminar Shear test of MWCNTs@PDA/PEI_(C)_; Table S7: All data of flexural test and Interlaminar Shear test of MWCNTs@PDA/PEI_(D)_.
