# Supplementary material for: Synchronous Improvement of Mechanical and Damping Properties of Structural Damping Composites with Polyetherimide Non-Woven Fabric Interlayers Loaded with Polydopamine and Carbon Nanotubes
Source: Polymers (Basel). 2023 Jul 21;15(14):3117. doi: 10.3390/polym15143117 (PMC10385600; doi:10.3390/polym15143117)
Supplement: Supplementary file 1 [file polymers-15-03117-s001.zip › polymers-2367782-supplementary.pdf]

Table. S1 All data of flexural test and Interlaminar Shear test of control

Control	Flexural strength/MP	Flexural modulus/GPa	Interlaminar Shear strength/MP
1	1723.41	122.25	96.27
2	1725.92	122.75	100.19
3	1727.45	124.22	104.14
4	1727.43	124.84	103.00
5	1726.28	125.08	106.93
6	1719.18	121.17	110.46
**Average value**	1724. 95	123.38	103.00
**Standard deviation value**	3.19	1.60	4.98

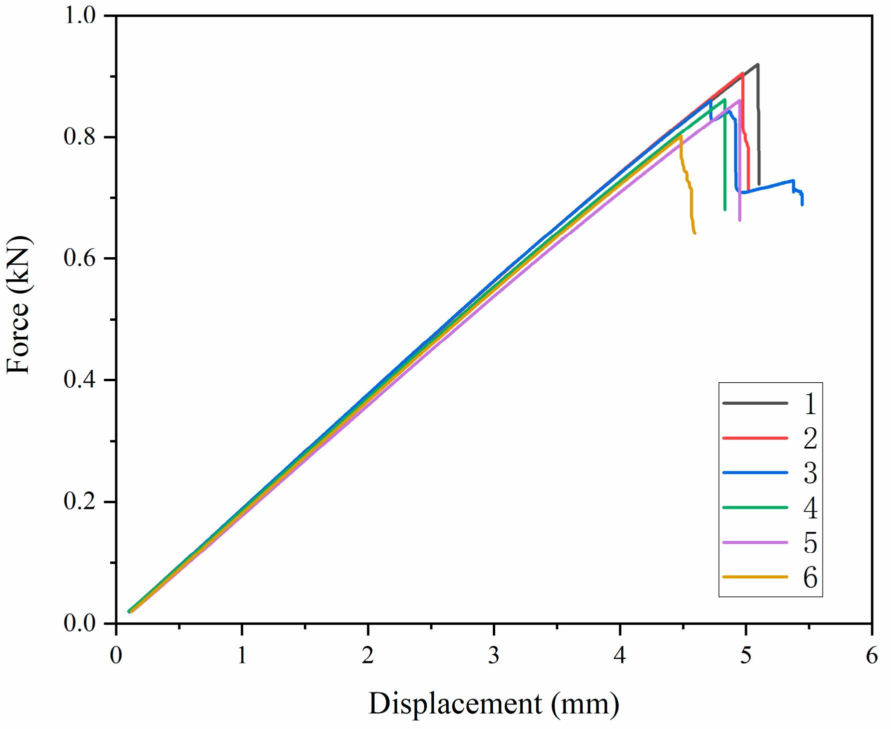

Fig.S1 Force-displacement curve of flexural test of control.

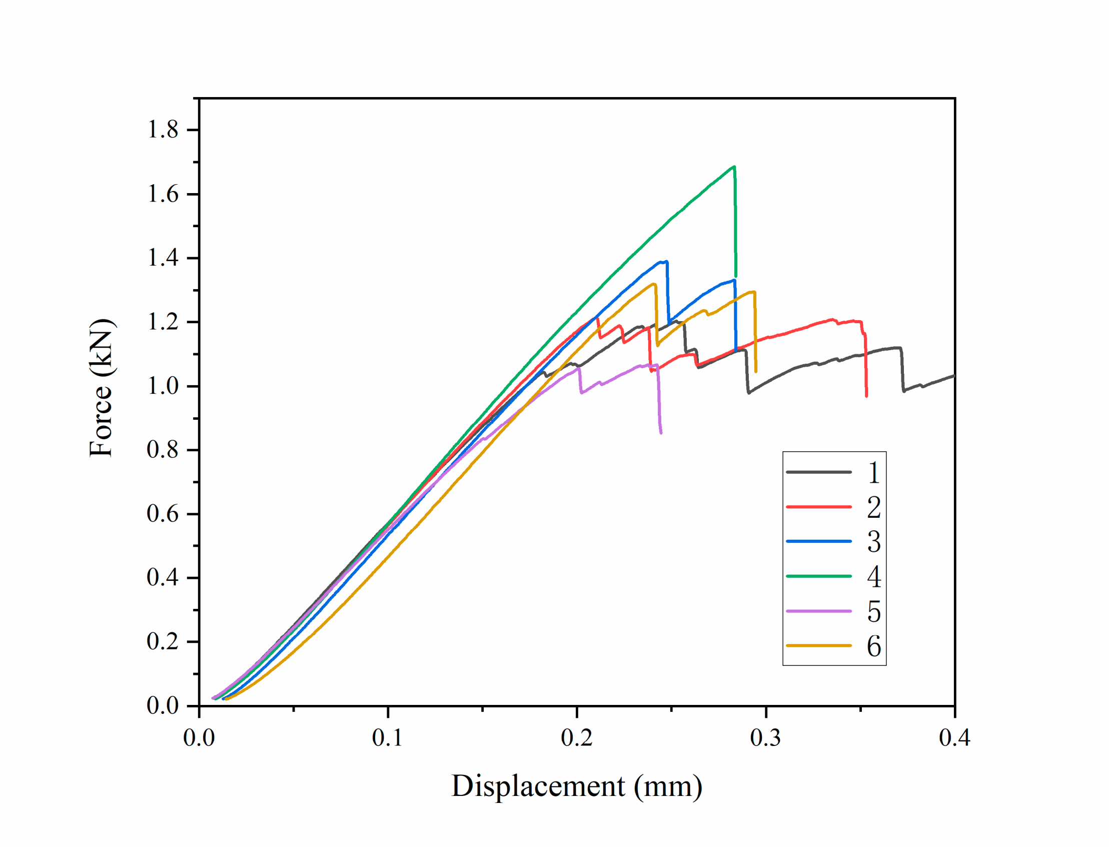

Fig.S2 Force-displacement curve of interlaminar shear strength test of control.

Table. S2 All data of flexural test and Interlaminar Shear test of PEI(C)

PEI(C)	Flexural strength/MP	Flexural modulus/GPa	Interlaminar Shear strength/MP
1	1607.35	116.63	101.84
2	1611.26	109.92	97.15
3	1613.92	111.49	103.68
4	1613.69	116.25	94.58
5	1611.83	108.92	98.49
6	1599.95	113.86	101.94
**Average value**	1609.67	112.84	99.61
**Standard deviation value**	5.32	3.25	3.50

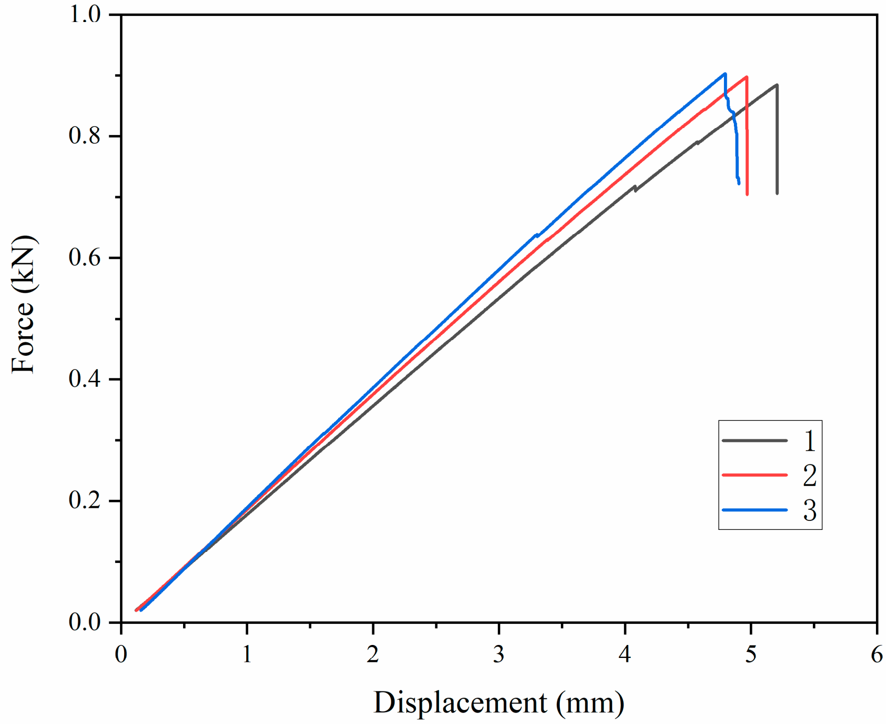

Fig.S3 Force-displacement curve of flexural test of PEI(C).

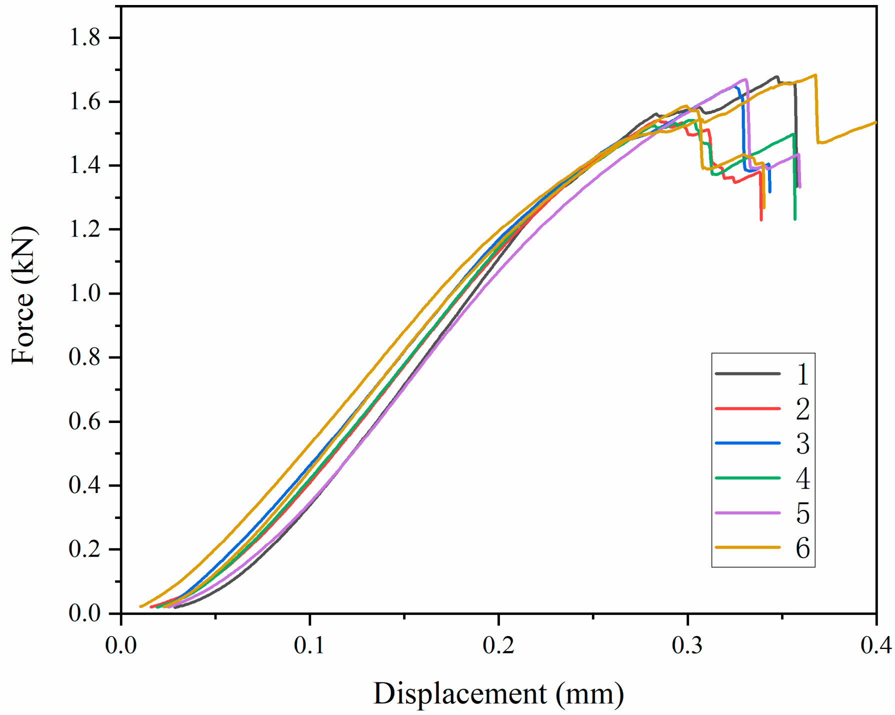

Fig.S4 Force-displacement curve of interlaminar shear strength test of PEI(C).

Table. S3 All data of flexural test and Interlaminar Shear test of $PEI_{(D)}$.

$PEI_{(D)}$	Flexural strength/MP	Flexural modulus/GPa	Interlaminar Shear strength/MP
1	1534.21	111.29	97.71
2	1536.21	109.70	94.34
3	1536.21	111.93	98.59
4	1532.38	110.45	92.37
5	1535.59	112.53	95.54
6	1542.68	111.39	90.05
**Average value**	1536.21	111.21	94.77
**Standard deviation value**	3.49	1.02	3.23

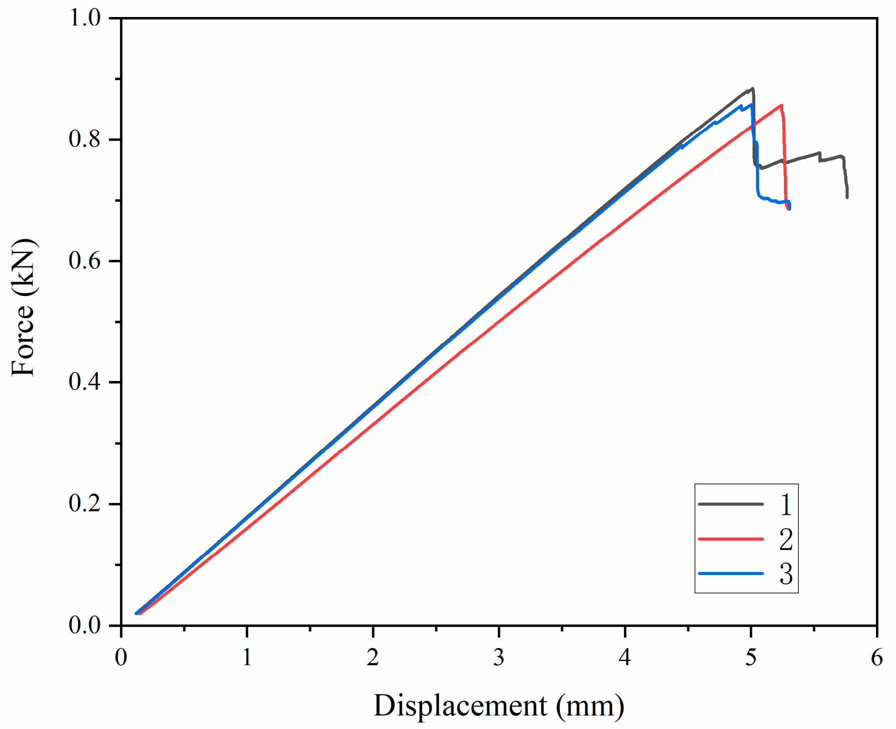

Fig.S5 Force-displacement curve of flexural test of $PEI_{(D)}$.

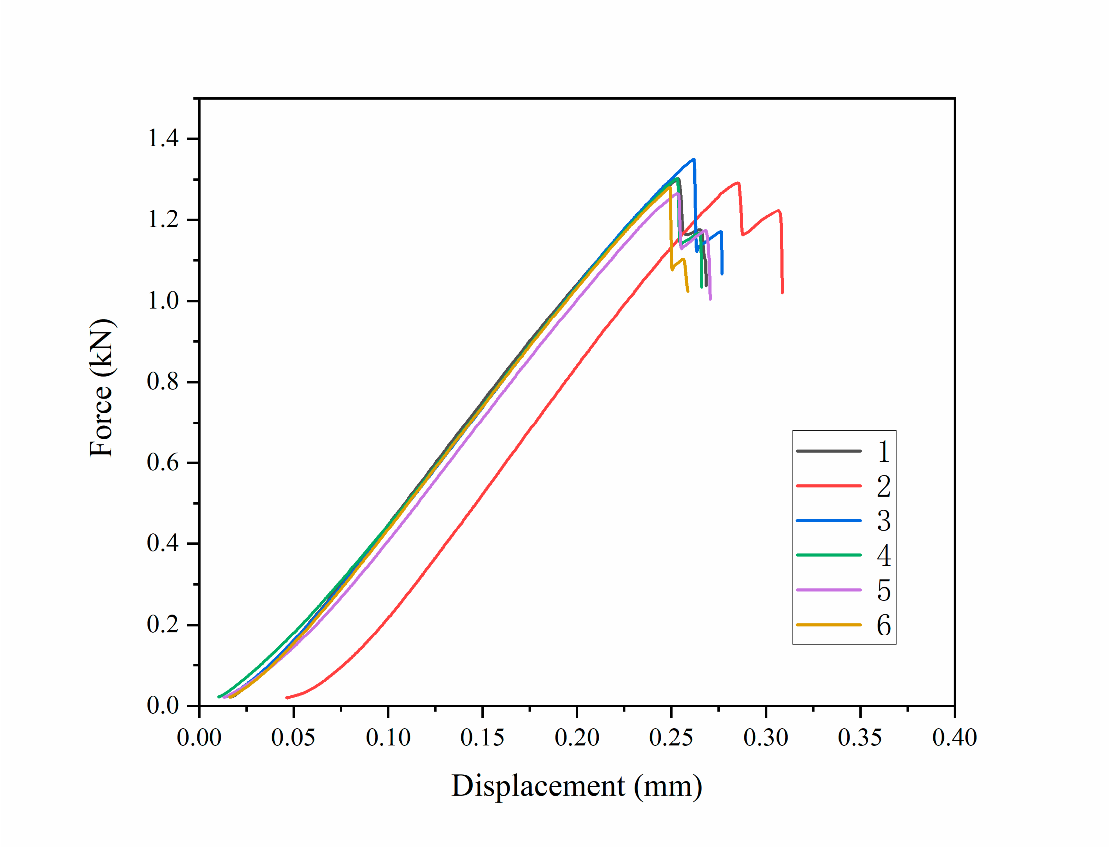

Fig.S6 Force-displacement curve of interlaminar shear strength test of PEI(D).

Table. S4 All data of flexural test and Interlaminar Shear test of MWCNTs/PEI(C).

**MWCNTs/PEI(C)**	**Flexural strength/MP**	**Flexural modulus/GPa**	**Interlaminar Shear strength/MP**
1	1676.75	127.80	108.61
2	1679.50	127.17	96.37
3	1680.87	127.42	99.71
4	1677.33	125.29	103.53
5	1672.05	127.15	97.66
6	1680.61	127.01	102.85
**Average value**	1677.90	126.97	101.46
**Standard deviation value**	3.30	0.87	4.49

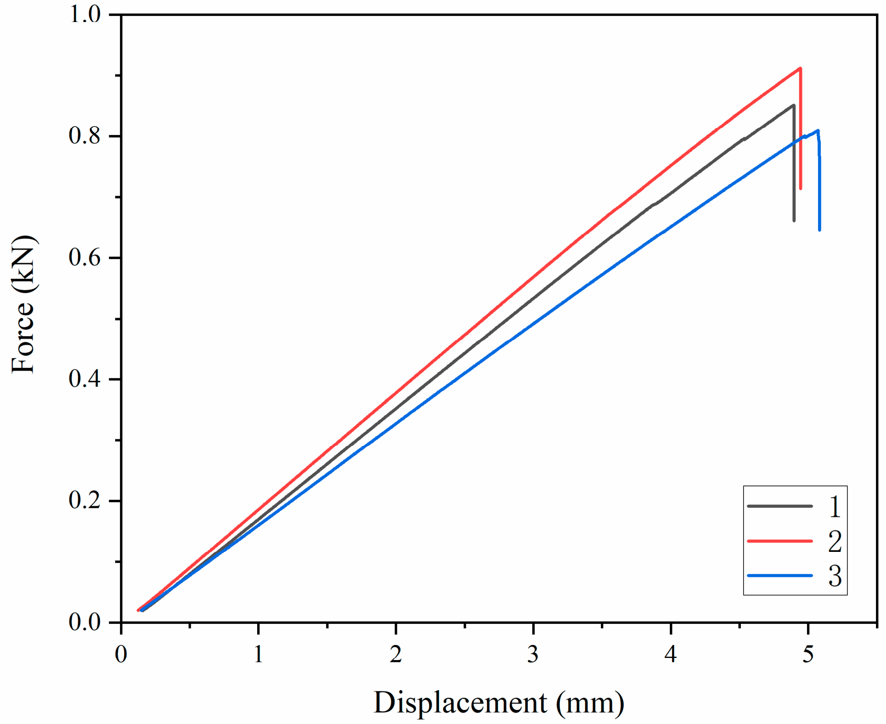

Fig.S7 Force-displacement curve of flexural test of MWCNTs/PEI(C).

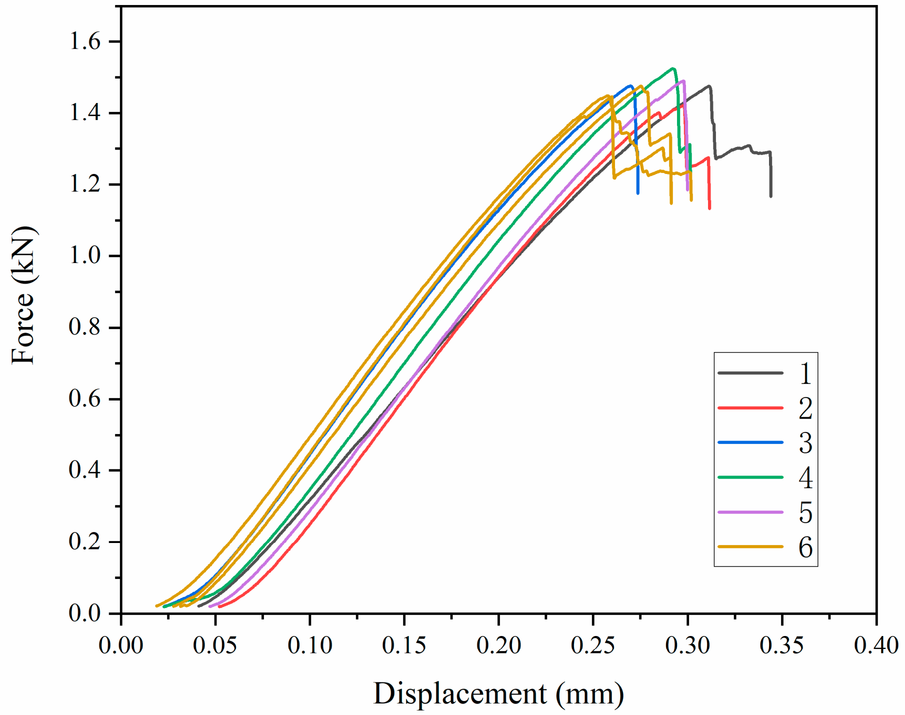

Fig.S8 Force-displacement curve of interlaminar shear strength test of MWCNTs/PEI(C).

Table. S5 All data of flexural test and Interlaminar Shear test of MWCNTs/PEI(D).

MWCNTs/PEI(D)	Flexural strength/MP	Flexural modulus/GPa	Interlaminar Shear strength/MP
1	1611.56	119.45	100.19
2	1621.64	120.13	101.95
3	1622.02	120.04	101.30
4	1614.35	119.44	99.91
5	1616.05	119.30	96.48
6	1617.78	119.56	105.17
**Average value**	1617.23	119.65	100.83
**Standard deviation value**	4.11	0.30	2.85

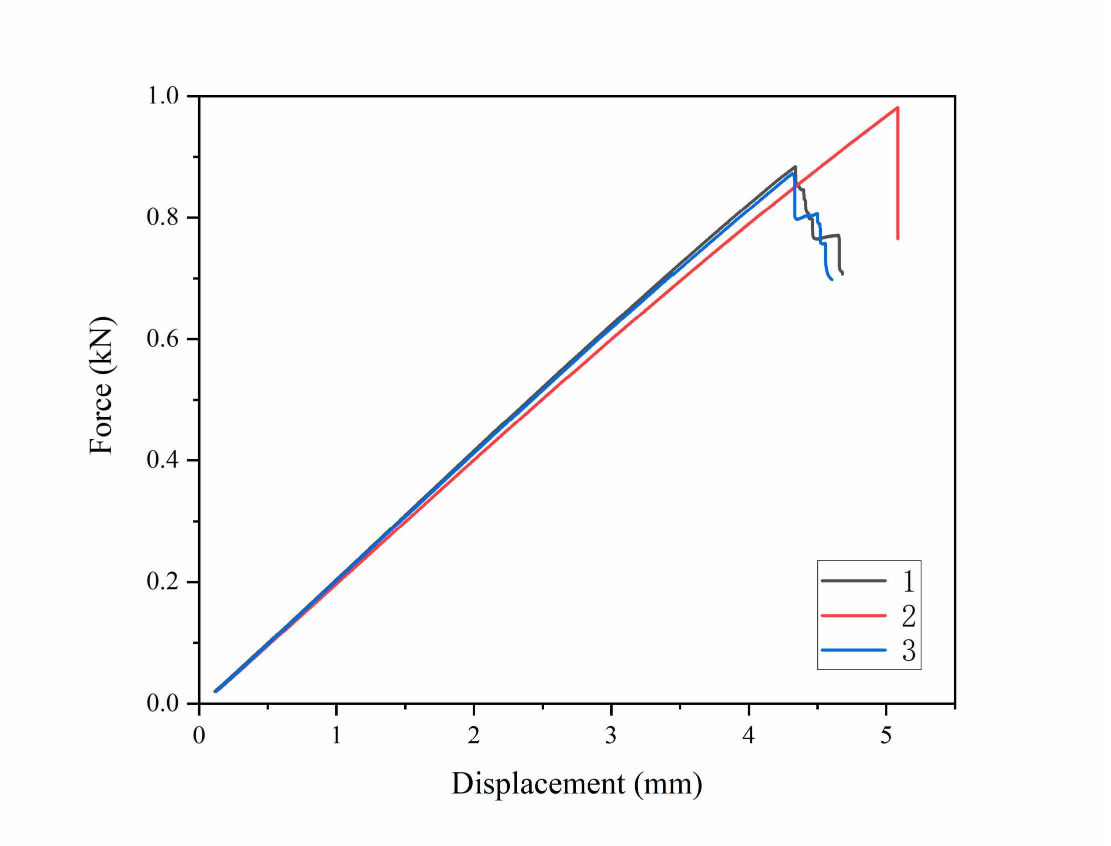

Fig.S9 Force-displacement curve of flexural test of MWCNTs/PEI(D).

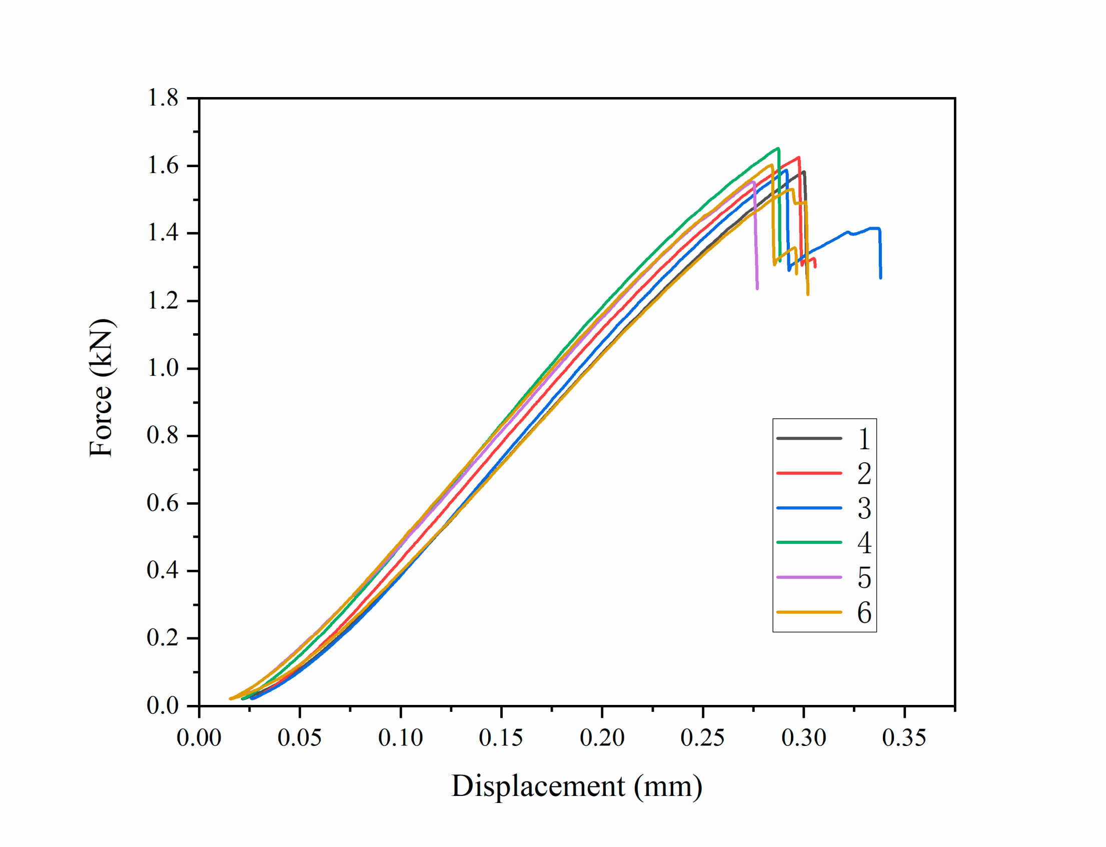

Fig.S10 Force-displacement curve of interlaminar shear strength test of MWCNTs/PEI(D).

Table. S6 All data of flexural test and Interlaminar Shear test of MWCNTs@PDA/PEI(C).

**MWCNTs@PDA /PEI(C)**	**Flexural strength/MP**	**Flexural modulus/GPa**	**Interlaminar Shear strength/MP**
1	1893.97	141.84	114.01
2	1899.60	141.84	107.83
3	1884.65	141.88	110.29
4	1885.04	145.81	110.22
5	1884.49	133.67	111.83
6	1885.35	145.97	107.58
**Average value**	1888.8	141.84	110.29
**Standard deviation value**	6.41	4.46	2.43

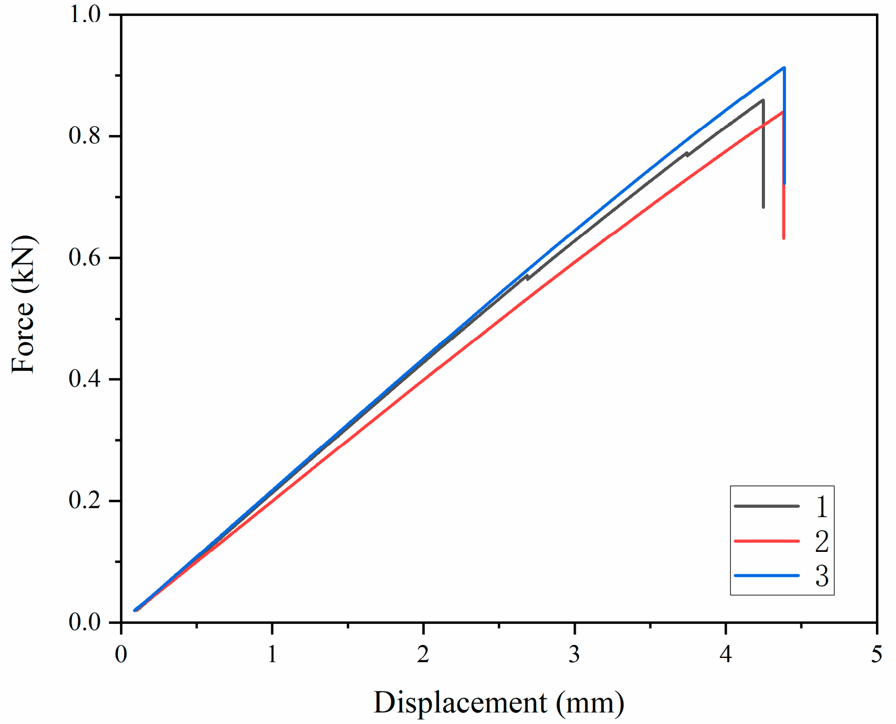

Fig.S11 Force-displacement curve of flexural test of MWCNTs@PDA/PEI(C).

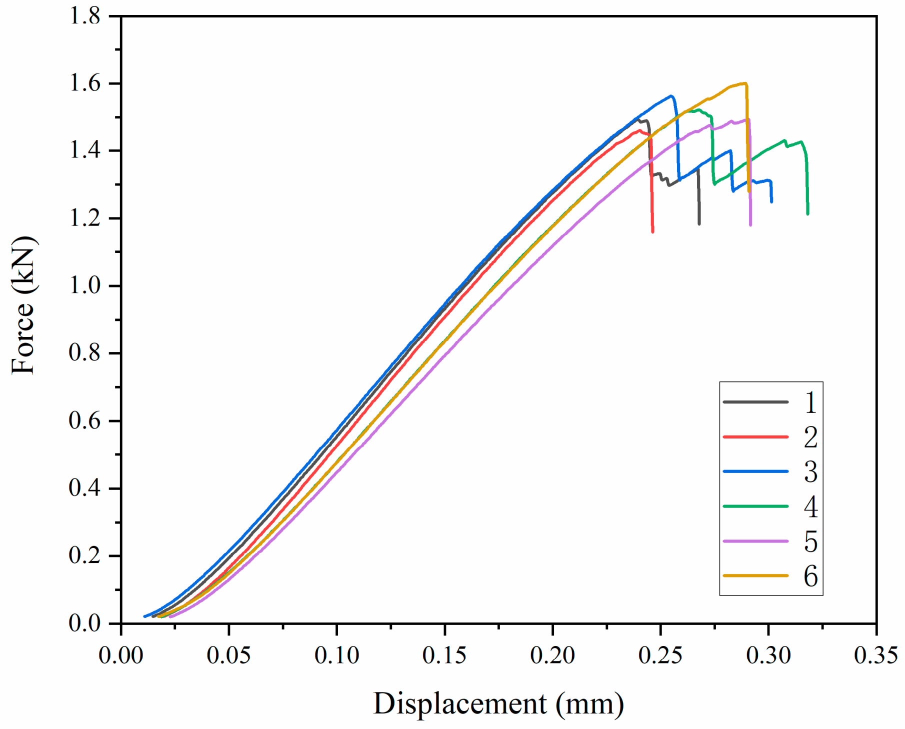

Fig.S12 Force-displacement curve of interlaminar shear strength test of

MWCNTs@PDA/PEI(C).

Table. S7 All data of flexural test and Interlaminar Shear test of MWCNTs@PDA/PEI(D).

MWCNTs@PDA/PEI(D)	Flexural strength/MP	Flexural modulus/GPa	Interlaminar Shear strength/MP
1	1868.91	133.34	104.38
2	1869.28	133.33	101.53
3	1864.58	136.63	107.17
4	1862.02	130.21	108.45
5	1869.29	133.30	115.04
6	1869.32	131.53	106.43
**Average value**	1867.23	133.06	107.17
**Standard deviation value**	3.16	2.16	4.55

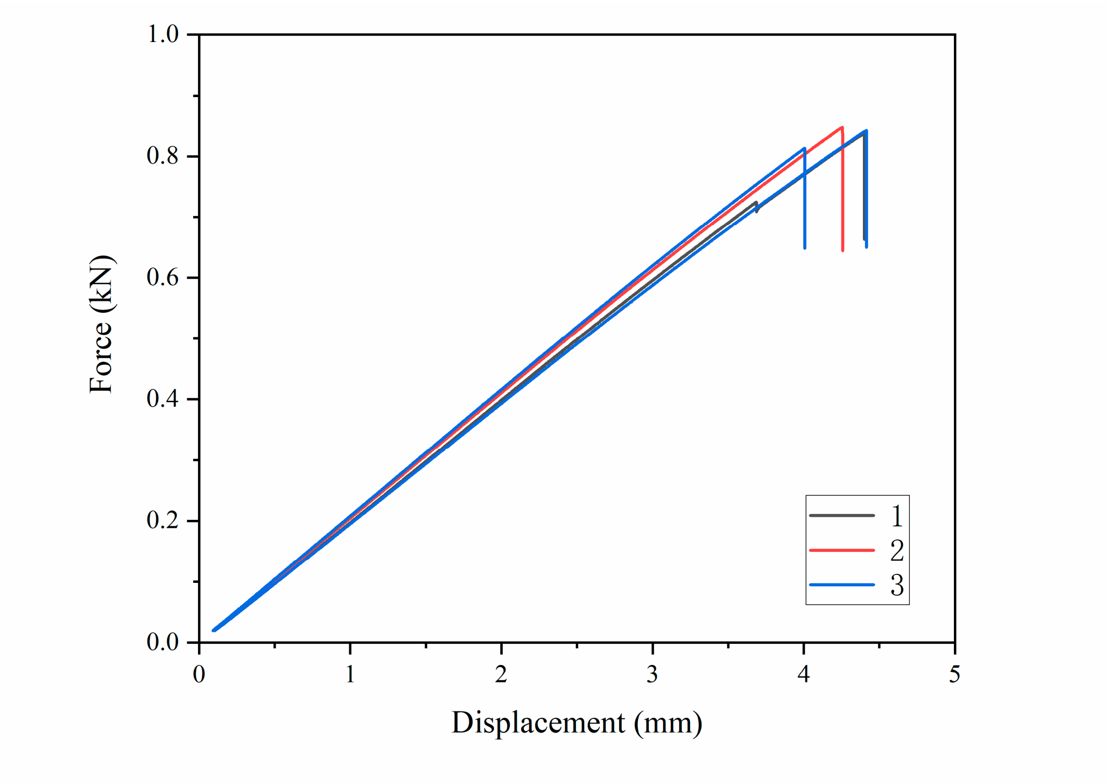

Fig.S13 Force-displacement curve of flexural test of MWCNTs@PDA/PEI(D).

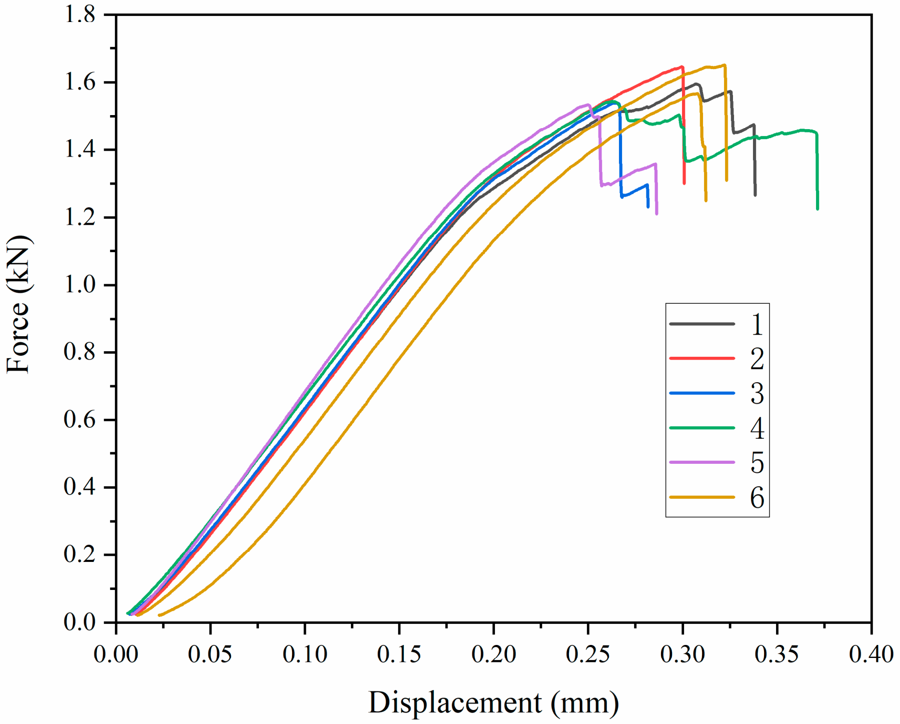

Fig.S14 Force-displacement curve of interlaminar shear strength test of MWCNTs@PDA/PEI(D).
